# Survival in canine tetanus – retrospective analysis of 42 cases (2006–2020)

**DOI:** 10.3389/fvets.2022.1015569

**Published:** 2022-12-15

**Authors:** Johannes Zitzl, Jens Dyckers, Arne Güssow, Hendrik Lehmann, Katarina Hazuchova

**Affiliations:** Department of Veterinary Clinical Sciences, Small Animal Clinic, Justus-Liebig-University Giessen, Giessen, Germany

**Keywords:** tetanus, canine, complication, prognosis, survival, respiratory, autonomic, severity class

## Abstract

**Objective:**

To define factors associated with survival in dogs with tetanus and to evaluate the prognostic significance of an established severity classification scheme.

**Methods:**

Medical records of dogs with the clinical diagnosis of tetanus were retrospectively reviewed with regard to signalment, clinical signs, clinicopathological findings on admission, wound characteristics, complications, therapeutic measures, and survival to discharge. Based on the extracted data, dogs were graded according to a previously published 4-class severity scheme. Non-parametric tests were applied for comparisons between survival categories.

**Results:**

Forty-two dogs fulfilled inclusion criteria, of which 32 survived. Of 10 non-survivors, 4 died and 6 were euthanised. Non-survivors were more often younger than 2 years of age (6/10 vs. 7/32 dogs, *p* = 0.023), had shorter duration of specific signs of tetanus (time from onset of typical signs to presentation) (2 vs. 4 days, *p* = 0.016), were prescribed less often antibiotics prior to presentation (*p* = 0.006), had higher tetanus severity grade (10/12 dogs in Class III or IV died, *p* < 0.001), more often received acepromazine (*p* = 0.009) and atropine (*p* = 0.012), and more often had hyperthermia (*p* = 0.005) and respiratory complications (pneumonia, laryngeal spasm; *p* = 0.008). Wound characteristics, the use of tube feeding, metronidazole, methocarbamol, magnesium and antitoxin were not significantly different between non-survivors and survivors.

**Clinical significance and conclusion:**

Young dogs with a rapid course of severe generalized tetanus have a guarded prognosis. The previously described severity classification scheme proved valuable in predicting survival. Prospective multi-center studies are needed to clarify the prognostic significance of age, sedative usage and modified versions of an established classification scheme, including the presence of respiratory complications.

## 1. Introduction

Tetanus is uncommon in dogs given their relative resistance to Clostridium tetani neurotoxin (1). Generalized forms prevail in this species, characterized by muscle rigidity, spasms and autonomic dysfunction in severe cases (2, 3). This is similar to clinical presentation of tetanus in humans, where complications arising from autonomic disturbances, aspiration pneumonia, hypoxaemia and sepsis represent leading causes of death (4–7). Mortality rates in humans are particularly high (33–92%) in low-income countries, with limited access to mechanical ventilation (8, 9), when compared to high-income countries (mortality 15–25%), where ventilatory support is available as needed (6, 10). In human patients with access to mechanical ventilation, the presence of autonomic dysfunction is the main predictor of poor outcome (5, 7).

In dogs, mortality rates between 8 and 50% (2, 3, 11–13) have been reported, with highest mortality in those developing autonomic (2) or respiratory complications, despite the use of mechanical ventilation or tracheostomy in some of these dogs (12). Nevertheless, a few reports of successful use of mechanical ventilation in canine tetanus can be found in the literature (3, 14).

Generally, given the low prevalence of tetanus in dogs, literature on this condition is scarce and leans partially on information about animals treated more than 20 years ago. Only one study, published in 2007, proposed a severity classification scheme associated with disease outcome (2). However, this scheme has not yet been assessed in an independent cohort of dogs with tetanus. As the standards of care in veterinary medicine likely have improved since this classification scheme was published, additional factors impacting outcome might be identified in cohorts including more recent cases. Such factors might not only be useful in outcome prediction but, depending on their nature, could also help guide treatment to improve survival.

Thus, the aim of the present study was to re-evaluate the prognostic significance of Burkitt et al.'s severity grading scheme (2), and to identify new factors associated with survival in dogs with tetanus. We hypothesized, that more severe disease as assessed by Burkitt et al.'s scheme and the presence of respiratory complications are associated with death in cases of canine tetanus.

## 2. Materials and methods

### 2.1. Study design, inclusion and exclusion criteria

A retrospective case series with analysis focusing on outcome (survival vs. non-survival) was conducted. For this purpose, the patient management system (easyVET^®^, VetZ GmbH, Isernhagen, Germany) at the Giessen University Small Animal Clinic was searched for the term “tetanus” in the records of dogs presented between 2006 and 2020. Dogs were included in the study based on characteristic signs of local or generalized tetanus at presentation, i.e., erect ears, risus sardonicus, wrinkled forehead and/or sawhorse stance. Dogs with a history or suspicion of ingestion of neurotoxic substances, significant ionized hypocalcaemia (≤ 0.8 mmol/L) (15) on admission, findings consistent with myositis, meningoencephalitis or spinal trauma, or missing essential medical data were excluded.

### 2.2. Medical records review

The following information was retrieved from medical records: signalment [including categorization of dogs as premature and mature (< 2 vs. > 2 years of age) as recently proposed (16)]; the month and season of presentation (October to March = cold season; April to September = warm season), also relating monthly distribution of cases to temperature and rainfall data (average from 1991 to 2020) collected by Germany's National Meteorological Service in the Giessen region; clinical signs observed by the owners prior to admission; clinical signs on admission including categories of body temperature [the lower/upper limit of normal temperature was set at 37.7/39.3 °C (17, 18)] and presence and localization of a wound; clinicopathological findings on admission (see below); and administered drugs and treatments (antibiotics, sedatives, muscle relaxants, assisted feeding). Treatment decisions were made based on the discretion of the attending clinician. Moreover, information about clinical deterioration during hospitalization (change of severity class based on the severity assessment scheme below) and complications were retrieved from the medical records. Complications were reported according to organ system: respiratory [aspiration pneumonia (based on radiographic evidence); laryngospasm (inspiratory dyspnoea with laryngeal stridor)], gastrointestinal {gastrointestinal dysmotility: delayed gastric emptying [< 4 peristaltic contractions per minute assessed with ultrasound (19)] and/or functional ileus [< 1 small bowel contractions per minute assessed with ultrasound (19)]; constipation [based on difficult evacuation of hard feces (20)]}, urogenital {dysuria [difficult and/or painful urinations (21)]; priapism} and other complications (not allocatable to any organ system). Where dogs fulfilled criteria for sepsis as previously defined [dysfunction of ≥ 2 organ systems in patient with suspected or confirmed infection (22)], this was recorded. However, this was not treated as a unique complication as it was considered to be the result of an underlying condition such as aspiration pneumonia. Finally, evidence of rapid eye movement (REM) sleep behavior disorders during hospitalization as recently defined (13), length of hospitalization and survival to discharge were recorded.

### 2.3. Severity assessment

Severity of disease on admission and during hospitalization was assessed retrospectively at the time of medical records review for the purpose of this study, using the 4-class scheme published by Burkitt et al. (2): Class I = facial signs compatible with tetanus (e.g., risus sardonicus, erect ears, enophthalmos) or hypersensitivity to light, noise or touch; Class II = generalized rigidity or dysphagia, with or without Class I findings; Class III = recumbency, tetanic spasms or seizures, with the prerequisite of having Class I or II signs; Class IV = abnormal heart rate or rhythm, blood pressure or respiration in addition to the Class III signs. For the purpose of severity classification, definition of bradycardia (≤ 60 beats/min), tachycardia (≥ 140 beats/min), bradypnoea (≤ 10 breaths/min), tachypnoea (≥ 40 breaths/min without panting), hypotension (systolic arterial blood pressure ≤ 80 mmHg) and hypertension (systolic arterial blood pressure ≥ 150 mmHg) were adopted from the study by Burkitt et al. (2) [a copy of the severity classification system of Burkitt et al. (2) is provided as Supplementary material (Supplementary 1)].

### 2.4. Laboratory findings

Hematology, biochemistry and blood gas analysis results obtained on the day of presentation or within the first 48 h thereafter were reviewed. Hematology was performed using either ADVIA^®^ 2120 (Siemens Healthcare GmbH, Erlangen, Germany) or ProCyte Dx (IDEXX GmbH, Kornwestheim, Germany), the latter being used when the dog presented out of hours. Heparinised plasma biochemistry was performed on ABX Pentra 400 (HORIBA ABX SAS, Montpellier, France) and blood gas analysis using cobas^®^ b 221 System (Roche Diagnostics Germany GmbH, Mannheim).

### 2.5. Treatment outcome

Treatment outcome was determined using the information provided in the medical records or by telephone follow-up in outpatients at the time of writing. Outcome was defined as negative or positive. Negative outcome was death related to tetanus, either spontaneous or by euthanasia (referred to as “non-survivors” throughout the manuscript). Positive outcome was survival to discharge in inpatients and uneventful recovery in outpatients (referred to as “survivors” throughout the manuscript).

### 2.6. Statistical analysis

Normality was assessed graphically (Q-Q plots and histograms) and by the means of statistical tests (Shapiro-Wilk test). Most of the data were not normally distributed, therefore, all data are expressed as median (range). Where the dataset was incomplete for a given parameter, then the proportion of those dogs for which data were available is presented. Non-parametric methods were applied for comparisons between non-survivors and survivors. Continuous variables were compared using the Mann-Whitney *U* test and categorical variables using either Chi-square or Fisher's exact test. *P* < 0.05 was considered significant. Statistical analysis was performed using IBM SPSS Statistics for Windows (Version 26.0. Armonk, NY, USA: IBM Corp.). Graphs were made in Microsoft^®^ Excel^®^ 2019 (version 2202, Microsoft Corporation, WA, USA).

## 3. Results

### 3.1. Study sample

Sixty-two dogs were identified by medical records search, of which 20 were excluded for the following reasons: tetanus suspected by the referring veterinarian, but not confirmed in the clinic (*n* = 5); tetanus discussed in the discharge summary as a possible differential, but a different final diagnosis made (*n* = 3); tetanus referred to in a different context (*n* = 2) (past history of tetanus in one dog, past vaccination against tetanus in another dog); primary neuromuscular disease or exposure to neurotoxic substances (*n* = 6); incomplete medical records (*n* = 4). Thus, 42 dogs were enrolled in the study. Nine dogs (21%) presented between 2006 and 2010, 14 dogs (33%) between 2011 and 2015 and 19 dogs (45%) between 2016 and 2020.

#### 3.1.1. Signalment

**Figure 1:**
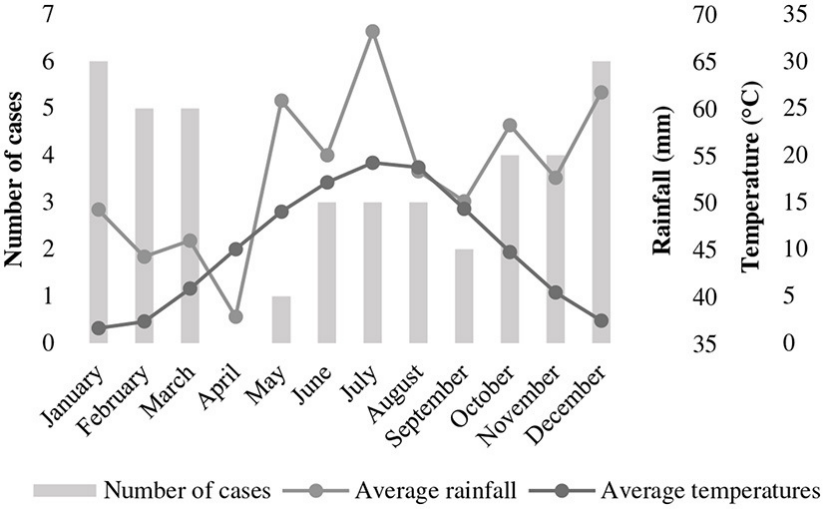
Distribution of cases of tetanus in 42 dogs presented to a university teaching hospital in Germany (2006–2020) across the months of the year in relationship to temperature and rainfall. Temperature and rainfall data represent the 30-year average (1991–2020) recorded in Gießen (Lahntal), Hessen, Germany [data extracted from Germany's National Meteorological Service online database (https://www.dwd.de/DE/leistungen/klimadatendeutschland/mittelwerte)].

Twenty breeds were represented: German Shepherd Dogs (*n* = 5), Labrador Retrievers (*n* = 4), Staffordshire Bullterriers (*n* = 3), Rhodesian Ridgebacks (*n* = 2) and one each of Malinois, Golden Retriever, American Staffordshire Terrier, American Bullterrier, Alaskan Malamute, German Hunting Terrier, Irish Terrier, German Shorthaired Pointer, Bernese Mountain Dog, Border Collie, Magyar Vizla, Doberman Pinscher, Weimaraner, Giant Schnauzer, Bernard Dog and Dogo Canario. Twelve dogs were cross-breed. Body weight ranged from 5.8 to 44 kg. Median age was 3 years (range, 0–9), with 31% (13/42) being younger and 69% (29/42) being older than 2 years. Half of the dogs were male and there were equal numbers of intact dogs of each gender (11/21 of each gender). Distribution of cases over the year was U-shaped, with 71% (30/42) of cases presenting during the cold season (Figure 1).

#### 3.1.2. Presenting complaints, physical examination findings and severity assessment

Tetanus specific symptoms were reported to be present for 3 days (range, 1–10) prior to presentation and in most instances progressed to the day of presentation. Wrinkled forehead (93%, 39/42), erect ears (88%, 37/42), stiffness / rigidity (81%, 34/42), risus sardonicus (71%, 30/42) and dysphagia (60%, 25/42) were most common, followed by trismus (41%, 17/42) and ocular abnormalities (36%, 15/42; strabismus in 12 dogs, enophthalmos / protrusion of the nictitating membrane in 6 dogs and miosis in 2 dogs). Fasciculations (10%, 4/42), hypersensitivity (10%, 4/42; noise and touch in 3 dogs and touch in 1 dog), stiff tail (7%, 3/42) and sawhorse stance (5%, 2/42) were less commonly reported and dysuria was reported in 1 dog (2%, 1/42) prior to presentation (4 more dogs developed dysuria during hospitalization as described in section 3.1.9 below). Five dogs (12%, 5/42) presented in lateral recumbency. Complaints other than those specific for tetanus were hypersalivation in 23 dogs (55%, 23/42), vomitus/regurgitation (it was generally unclear, which one had occurred) and cough in 12 dogs each (29%, 12/42 each) and diarrhea in 3 dogs (7%, 3/42).

Included dogs had a median 5-point body condition score value of 3 (range, 2–4). Ninety-three percent (39/42) of the dogs had normal heart rate, and 3 dogs (7%, 3/42) had tachycardia (≥ 140/min). Nine dogs (21%, 9/42) presented with tachypnoea (≥ 40/min) and two dogs (5%, 2/42) with spasm-driven labored respiration. Seven dogs (17%, 7/42) had body temperature above the upper limit (> 39.3 °C; range, 39.4–40.9), but none was hypothermic (< 37.7 °C). ECG was performed in 3 dogs initially and was abnormal in one dog (second degree atrioventricular block).

Using the classification scheme of Burkitt et al. (2), 8 dogs (19%, 8/42) initially fitted the criteria for tetanus severity Class I, 28 dogs (67%, 28/42) for Class II, 3 dogs (7%, 3/42) for Class III and 3 dogs (7%, 3/42) for Class IV. Inclusion into the Class IV severity category on admission was in all cases made based on the presence of tachycardia. Of the 39 hospitalized dogs (3 dogs were treated on an outpatient basis), 7 (18%) progressed to a higher severity Class during the course of their disease. One dog progressed from Class II to III, 5 progressed from II to IV, and 1 from III to IV. Overall, the worst/highest achieved severity class was Class I in 6, Class II in 21, Class III in 3 and Class IV in 9 of the 39 hospitalized dogs. For the 6 dogs who progressed to Class IV during hospitalization, the criterion used to assign them to Class IV was respiratory arrest in 3 dogs (1 of those 3 had systemic hypotension as well), tachycardia in 2 and systemic hypertension in 1.

Disturbances in the REM sleep were noted in 5 dogs from Classes I (*n* = 1), II (*n* = 3) and III (*n* = 1). Signs comprised paddling / running movements (*n* = 4), fasciculations (*n* = 3), vocalization (*n* = 2) and jaw chomping (*n* = 1).

#### 3.1.3. Wound characteristics

Sixty-seven percent (28/42) of the dogs had a history of a wound identified by veterinarian/owner. The median interval from the injury / the time the wound was first observed to the onset of clinical signs of tetanus was 7 days (range, 2–39). Twenty-six wounds as reported by the owners could be confirmed and another 8 wounds, not noted by the owner / referring veterinarian, could be detected during initial examination. Three dogs had 2 wounds concurrently, making 34 wounds affecting 31 dogs (74%) upon admission. Twenty-six wounds (76%, 26/34) were located at distal extremities (18 front limb; 8 pelvic limb; location within the extremities: 24 digital; 1 footpad; 1 metatarsal), 4 were related to the oral cavity / head (3 dental; 1 healing buccal mucous membrane laceration associated with ipsilateral mandibular lymphadenitis), 2 were related to the abdomen (1 post ovariohysterectomy; 1 bite wound) and 2 were located to the thorax [1 suspicion of esophageal trauma (based on the presence of cervical emphysema and radiological evidence of pneumomediastinum); 1 injury by a wooden stick on the lateral aspect of the thorax not penetrating into the thoracic cavity].

Surgical wound debridement was performed in 14 dogs after a median of 16 h (range, 2–42) of hospitalization. Of the 11 dogs (26%, 11/42) where no wound could be identified, one had flea infestation.

#### 3.1.4. Clinicopathological findings

Hematological and clinical chemistry findings were available for review in 39 (93%, 39/42) and 36 dogs (86%, 36/42), respectively. Ninety-five percent of the hematology and 56% of the chemistry results stemmed from the day of initial presentation at our institution. Half of the dogs (21/42) had venous blood gas analysis performed upon initial presentation, including lactate measurements in all but one dog.

With the exception of creatine kinase activity (CK), which was abnormal in 59% (17/29) of cases where this parameter was measured [median, 783 U/L (range, 229–24,680 U/L)], there were no or only minimal derangements of the remaining laboratory parameters, considered not to be of clinical relevance. Some findings could be attributed to the young age (< 6 months).

#### 3.1.5. Antibiotics

Sixty-seven percent (28/42) of the dogs were treated with antibiotics prior to presentation, and in 12 more dogs antibiotics were started upon admission, leaving 2 dogs (5%, 2/42) without antibiotic treatment for reasons unknown. Prior to admission, amoxicillin with or without clavulanate was prescribed most often (61%, 17/28), followed by penicillin (29%, 8/28), streptomycin (18%, 5/28), fluoroquinolones (18%, 5/28), metronidazole (11%, 3/28), clindamycin (11%, 3/28) and doxycycline (4%, 1/28). Prior to referral, 10 dogs were treated with more than one antibiotic. From the time of presentation, metronidazole (78%, 31/40) was the most often used antibiotic, followed by amoxicillin/clavulanic acid (60%, 24/40), ampicillin (15%, 6/40) and fluoroquinolones (13%, 5/40). Eighteen (45%, 18/40), 18 (45%, 18/40) and 4 dogs (10%, 4/40) received 1, 2 and 3 antibiotics, respectively. Combination of metronidazole and amoxicillin/clavulanate was the most common reason for the use of 2 antibiotics concurrently (*n* = 11). Five dogs received 2 antibiotics because of perioperative ampicillin usage and 1 dog received ampicillin perioperatively and 2 other antibiotics. Evidence of sepsis was the reasons for concurrent administration of 3 antibiotics in one dog, in 3 more dogs the reason was unknown. In the remaining dogs the reason for administration of more than one antibiotic could not be identified from medical records.

#### 3.1.6. Sedation, muscle relaxation and atropine use

Thirty-six dogs (86%, 36/42) received sedatives, whereby benzodiazepines were used most often (81%, 34/42), followed by acepromazine (50%, 21/42), butorphanol (31%, 13/42) and barbiturates (14%, 6/42). Twenty-eight dogs (67%, 28/42) received two or more sedatives at the same time. Dosage of sedatives as extracted from medical records was as follows (number of dogs for which respective information was available and mode of administration are indicated in square brackets): midazolam 4.2 mg/kg/day {range, 2.1–12.1 mg/kg/day; [*n* = 21, intravenous (IV) bolus and/or constant rate infusion (CRI)]}; diazepam 1.3 mg/kg/day {range 0.7–3.2 mg/kg/day; [*n* = 13, peroral (PO) and/or IV bolus]}; tetrazepam 4.3 mg/kg/day [range, 3.3–5.3 mg/kg/day; (*n* = 2, PO)]; acepromazine 0.3 mg/kg/day [range, 0.02–1.9 mg/kg/day; (*n* = 13, IV bolus)]; butorphanol 2.8 mg/kg/day [range, 0.4–11.0 mg/kg/day; (*n* = 9, IV bolus and/or CRI)]; phenobarbital 3.3 mg/kg/day [range, 1.0–11.8 mg/kg/day; (*n* = 3, PO and/or IV bolus)]; pentobarbital 3.4 mg/kg/day [*n* = 1, CRI].

Methocarbamol was prescribed for 22 dogs (52%, 22/42), of which 21 received benzodiazepines concurrently. Dosage of methocarbamol was recorded in 21 cases and ranged from 72 to 310 mg/kg/day (median, 154 mg/kg/day). Application routes of methocarbamol were PO, IV and/or CRI. Magnesium was given in 33 cases (79%, 33/42) PO and/or parenterally (as a supplement of crystalloid IV fluids).

Atropine was administered as IV bolus injection and/or *via* CRI in 18 dogs (43%, 18/42).

#### 3.1.7. Tetanus antitoxin

In 5 dogs tetanus antiserum (1 of human origin, 4 equine) was administered by the referring veterinarian prior to presentation, another 19 dogs received equine antitoxin during hospitalization [in total, 57% (24/42) dogs received tetanus antitoxin]. Information regarding time from onset of tetanic signs to antitoxin administration was available in 17 dogs and was 97 h (range, 25–149). The dose of antiserum administered was known in 18 dogs and ranged from 86 to 1,666 U/kg (median, 357 U/kg). In a descending order, route of administration of tetanus immunoglobulin was IV (*n* = 14), subcutaneous (*n* = 3) and intramuscular (*n* = 1). Adverse reactions to antitoxin were not reported.

#### 3.1.8. Tube feeding

In 57% of the dogs (24/42) nutrition was temporarily provided *via* feeding tube. Seventeen dogs had a percutaneous endoscopic gastrostomy (PEG) tube placed and the remainder (7/24) received an oesophagostomy (4/7) or a nasogastric tube (3/7). Information regarding time to initiation of assisted enteral feeding was available for all but one dog and ranged from 2 to 72 h (median, 19) after admission.

#### 3.1.9. Complications

Fifteen dogs (38%, 15/39) had complications, whereby 6 dogs had more than one. Respiratory complications occurred in 5 dogs and included laryngospasm in 1 (necessitating endotracheal intubation with the aid of a stylet and short-term mechanical ventilation; the indication for the mechanical ventilation could not be discerned based on medical records) and aspiration pneumonia in 4 dogs. Gastrointestinal complications occurred in 8 dogs, including gastrointestinal dysmotility in 6 and constipation in 2 dogs. Genitourinary complications comprised dysuria in 5 and priapism in 2 dogs. Other/unclassified complications were percutaneous endoscopic gastrostomy (PEG) insertion site infection (*n* = 1) and tongue trauma (due to trismus, necessitating surgery; *n* = 1). One dog with severe gastrointestinal dysmotility and 2 dogs with both aspiration pneumonia and gastrointestinal dysmotility had evidence of sepsis.

#### 3.1.10. Survival, reasons for death/euthanasia and length of hospitalization

Thirty-two dogs (76%, 32/42) survived to discharge. Of these, 3 were managed on an outpatient basis. Of the 10 dogs not surviving to discharge, 4 died and 6 were euthanised due to severity of the disease and lack of owner commitment to further treatment when initial improvement was not seen (*n* = 4) or financial constraints (*n* = 2).

Reasons for death (as opposed to euthanasia) were cardiovascular failure preceded by respiratory arrest in 3 dogs, and 1 dog died of septic shock. Cardiopulmonary resuscitation was unsuccessful in all 3 dogs with respiratory arrest and was declined in the dog dying of septic shock.

None of the Class IV and only 2/3 of the Class III dogs survived. Time of hospitalization in survivors with respect to disease severity was 4 days (range, 2–8) in Class I; 8 days (range, 2–28) in Class II; 12 days (range, 11–13) in Class III.

### 3.2. Comparisons between survival categories

#### 3.2.1. Age, gender, and neutering status

Non-survivors were significantly more often younger than 2 years, but there was no effect of sex and neutering status on survival (Table 1).

**Table 1:** Differences in age, gender and neutering status between survival categories in 42 dogs with tetanus.

**Variable**	**Category**	**Non-survivors No. (%)**	**Survivors No. (%)**	***P*-value**
Age (y)	< 2	6 (60)	7 (22)	**0.023**
	> 2	4 (40)	25 (78)	
Gender	Female	7 (70)	14 (44)	0.147
	Male	3 (30)	18 (56)	
Neutering	Entire	6 (60)	16 (50)	0.580
status	Neutered	4 (40)	16 (50)	
Total		10 (100)	32 (100)	

No., Number; y, years.

Bold values denote statistical significance at the *P* < 0.05 level.

#### 3.2.2. Presenting complaints, physical examination findings, wound location, and disease severity

When compared to survivors, non-survivors had a significantly shorter duration of clinical signs compatible with tetanus [median, 2 (range, 1–4) vs. 4 (range, 1–10) days, *p* = 0.016] and were more likely to show trismus upon admission (Table 2). Non-survivors also had significantly more often history of vomiting/regurgitation prior to admission and had significantly more often hyperthermia and tachycardia on initial physical examination, while there was no difference in other anamnestic and physical examination parameters, including wound location (Table 2).

**Table 2:** Differences in anamnestic data, baseline clinical signs and disease severity between survival categories in 42 dogs with tetanus.

**Variable**	**Category**	**Non-survivors** **No. (%)**	**Survivors No. (%)**	***P*-value**
History of vomiting/regurgitation	No	4 (40)	26 (81)	**0.012**
	Yes	6 (60)	6 (19)	
History of coughing	No	8 (80)	22 (69)	0.492
	Yes	2 (20)	10 (31)	
History of hypersalivation	No	4 (40)	15 (47)	0.703
	Yes	6 (60)	17 (53)	
Trismus upon admission	No	2 (20)	23 (72)	**0.004**
	Yes	8 (80)	9 (28)	
BCS upon admission	Ideal	7 (70)	29 (91)	0.116
	Underweight	3 (30)	2 (6)	
	Overweight	0 (0)	1 (3)	
Rectal temperature upon admission	Normal	6 (60)	29 (91)	**0.023**
	Hyperthermia	4 (40)	3 (9)	
	Hypothermia	0 (0)	0 (0)	
Respiratory rate upon admission	Normal	8 (80)	25 (78)	0.900
	Tachypnoea	2 (20)	7 (22)	
	Bradypnoea	0 (0)	0 (0)	
Heart rate upon admission	Normal	7 (70)	32 (100)	**0.001**
	Tachycardia	3 (30)	0 (0)	
	Bradycardia	0 (0)	0 (0)	
Wound location^a^	No wound	3 (30)	8 (26)	0.119
	Extremities	4 (40)	21 (68)	
	Oral cavity	1 (10)	2 (6)	
	Other location	1 (10)	0 (0)	
Tetanus severity grading^b^ upon admission	Class I	0 (0)	8 (25)	**0.001**
	Class II	5 (50)	23 (72)	
	Class III	2 (20)	1 (3)	
	Class IV	3 (30)	0 (0)	
Progression to worse severity class during the course of the disease^b, c^	No	4 (40)	28 (97)	**< 0.001**
	Yes	6 (60)	1 (3)	
Total		10 (100)	32 (100)	

BCS, body condition score; No., Number.

^a^Excluding 1 dog with both an oral and a digital wound.

^b^According to Burkitt et al. (2).

^c^Excluding 3 dogs treated on an outpatient basis.

Bold values denote statistical significance at the *P* < 0.05 level.

Regarding assignment to severity classes according Burkitt et al. (2), non-survivors significantly more often were assigned a worse severity class upon admission. While all but 1 survivor presented Class I or II signs only, 50% of the non-survivors presented in Class III or IV (Table 2). Furthermore, non-survivors significantly more often deteriorated during hospitalization (switched to a worse/higher severity class) (Table 2).

#### 3.2.3. Wound debridement, antibiotics, sedatives, muscle relaxants, antitoxin, atropine, and tube feeding

Comparison of treatment modalities between survival categories is presented in Table 3.

**Table 3:** Differences in treatment between survival categories in 42 dogs with tetanus.

**Variable**	**Category**	**Non-survivors No. (%)**	**Survivors No. (%)**	***P*-value**
Surgical wound debridement	No	5 (50)	22 (69)	0.280
	Yes	5 (50)	10 (31)	
Metronidazole	No	3 (30)	8 (25)	0.754
	Yes	7 (70)	24 (75)	
Amoxicillin-Clavulanate	No	2 (20)	16 (50)	0.094
	Yes	8 (80)	16 (50)	
Ampicillin	No	8 (80)	28 (88)	0.554
	Yes	2 (20)	4 (13)	
Fluroquinolones^a^	No	8 (80)	29 (91)	0.365
	Yes	2 (20)	3 (9)	
Antibiotic other than metronidazole only	No	8 (80)	25 (78)	0.900
	Yes	2 (20)	7 (22)	
Benzodiazepines^b^	No	1 (10)	7 (22)	0.404
	Yes	9 (90)	25 (78)	
Acepromazine	No	1 (10)	20 (63)	**0.004**
	Yes	9 (90)	12 (38)	
Barbiturates^c^	No	6 (60)	30 (94)	**0.008**
	Yes	4 (40)	2 (6)	
Sedatives^d^	0	1 (10)	5 (16)	0.106
	1	0 (0)	8 (25)	
	2	1 (10)	7 (22)	
	≥ 3	8 (80)	12 (38)	
Methocarbamol	No	4 (40)	16 (50)	0.580
	Yes	6 (60)	16 (50)	
Magnesium	No	1 (10)	8 (25)	0.313
	Yes	9 (90)	24 (75)	
Atropine	No	2 (20)	21 (66)	**0.008**
	Yes	8 (80)	10 (31)	
Antitoxin^e^	No	4 (40)	12 (38)	1
	Yes	6 (60)	18 (56)	
Tube feeding^f^	No	2 (20)	16 (50)	0.094
	Yes	8 (80)	16 (50)	
Total		10 (100)	32 (100)	

Treatments are listed as recorded in the patient management system, irrespective of indication for their use.

No., Number.

^a^Enrofloxacin or Marbofloxacin.

^b^Midazolam, Diazepam or Tetrazepam.

^c^Phenobarbital or Pentobarbital.

^d^Benzodiazepines, Barbiturates, Acepromazine or Butorphanol.

^e^Humane or equine immunoglobulin, administered prior to or in the course of hospitalization.

^f^Nasogastric,tube, esophagostomy tube or percutaneous endoscopic gastrostomy (PEG) tube.

Bold values denote statistical significance at the *P* < 0.05 level.

Neither performing surgical debridement nor time from presentation until surgery [non-survivors: 17 h (range, 3–88); survivors: 19 h (range, 2–184); *p* = 0.930] had an effect on survival. Non-survivors received significantly less often antibiotics prior to presentation (3/10 non-survivors vs. 25/32 survivors received antibiotics prior to presentation, *p* = 0.006), but the choice of a particular antibiotic or omission of metronidazole from treatment regime did not impact survival.

Non-survivors more often received acepromazine, barbiturates and atropine. There was no significant difference between non-survivors and survivors with respect to the number of administered sedatives, use of methocarbamol and use of magnesium.

Non-survivors received antitoxin earlier in the course of their disease [50 h (range, 26–51) vs. 99 h (range, 25–149), *p* = 0.006], but the use of antiserum was not associated with improved survival rate. Furthermore, neither feeding tube placement nor time to starting enteral feeding [17 h (range, 3–65) vs. 20 h (range, 2–72), *p* = 0.636] differed significantly between non-survivors and survivors.

#### 3.2.4. Complications

Non-survivors more often had respiratory complications, whereas frequency of complications in other organ systems did not differ between survival categories (Table 4).

**Table 4:** Differences in complications between survival categories in 39 dogs with tetanus treated as inpatients.

**Complication**	**Category**	**Non-survivors No. (%)**	**Survivors No. (%)**	***P*-value**
Respiratory^a^	No	6 (40)	28 (97)	**0.008**
	Yes	4 (60)	1 (3)	
Gastrointestinal^b^	No	6 (60)	25 (86)	0.077
	Yes	4 (40)	4 (24)	
Urogenital^c^	No	9 (90)	23 (79)	0.448
	Yes	1 (10)	6 (21)	
Other^d^	No	10 (100)	27 (93)	0.394
	Yes	0 (0)	2 (7)	
Total		10 (100)	29 (100)	

No., number.

^a^Aspiration pneumonia, laryngospasm.

^b^Delayed gastric emptying and/or functional ileus, constipation.

^c^Dysuria, priapism.

^d^Percutaneous endoscopic gastrostomy (PEG) insertion site infection, tongue trauma due to trismus.

Bold values denote statistical significance at the *P* < 0.05 level.

## 4. Discussion

This study aimed at searching for factors, that might be of relevance to survival in dogs with tetanus. Several such factors were identified, most importantly younger age, duration of clinical signs, disease severity based on classification by Burkitt et al. (2), disease progression and respiratory complications. The study could confirm the validity and usefulness of Burkitt et al.'s severity classification scheme and introduced the concept of categorizing complications by organ systems. Eventually, while findings of this study advocate the use of antibiotics early in the course of disease, choice of one of the recommended antibiotic compounds (1) seems to be of minor significance in terms of survival as does wound debridement and antitoxin usage.

Generally, tools using clinical signs to assess severity of disease are extremely useful in clinical practice settings as they can be applied by any veterinarian and do not require special equipment. It was therefore one of our aims to re-assess the validity of Burkitt et al.'s classification scheme (2) in this independent cohort of dogs with tetanus, separated by both space and time from Burkitt et al.'s cohort. Confirming the universal validity of Burkitt et al.'s scheme, it proved useful in terms of prognostication in the present study too. All 9 Class IV dogs died as did 1/3 Class III dogs, while all Class I and II dogs survived. In Burkitt at al's cohort, 7/14 Class IV dogs and 1/5 Class III dogs did not survive, and no death was reported for dogs in Classes I and II. Although the mortality rate of Class IV dogs in Burkitt et al.'s study was lower than in the present report, the higher euthanasia rate in our study (5/9 Class IV dogs) when compared to Burkitt et al.'s cohort (2/14 Class IV dogs) represents an important confounder. Indeed, the overall mortality rate is comparable between our [10/42 (24%)] and the former report [8/35 (23%)]. This is an interesting finding, suggesting that mortality rates in dogs with tetanus have not improved over the past 15 years.

Besides evaluation of the prognostic relevance of this previously published severity classification scheme (2), this study aimed to identify additional factors associated with survival, which can easily be assessed by clinicians upon initial presentation such as history, signalment and physical examination findings. In this regard, the association of non-survival with younger age, history of vomiting/regurgitation, shorter duration of the disease and the presence of trismus might be promising and should be assessed as potential prognostic factors in prospective studies.

Tetanus is more prevalent in younger dogs who also have previously been reported to suffer from a more severe disease (2). It has been suggested that this age-related susceptibility might be associated with immune system status or increased environmental exposure with advancing age (1). In the present study younger age (< 2 years) was associated with non-survival. Similarly, older age as a factor of improved survival has been documented in equine tetanus as well (23).

Although not evaluated for its prognostic significance, but not less important for the veterinary community is the seasonality of tetanus. A higher frequency of cases in the UK during the cold season was recently documented by Starybrat et al. (24), and was also identified in the present report. This seasonality might be due to the exposure to wet and muddy conditions in winter and fall, which predisposes to increased contamination of wounds with soil-containing spores (24). Seasonality, however, seems to be dependent on uniformity of climate (as opposed to extremes) as was recently proposed in a study of dogs with tetanus from California, USA, where no such seasonality exists (25). At least in the moderate climate zones of Europe, clinicians should bear in mind, that tetanus is more prevalent during cold months and put the disease on their list of possible differentials. Although tetanus usually has typical clinical presentation, some cases present with vague symptoms and ocular abnormalities. Ocular abnormalities such as swollen eyelids, protrusion of the third eyelid / enophthalmos, epiphora, and strabismus might represent the only clinical signs in the early stage of the disease (2, 26), and there is some risk the disease might initially go unrecognized by less experienced clinicians.

Vomiting has been infrequently reported in dogs with tetanus, either as a vague early sign of the disease (2, 27) or as a complication of PEG-tube feeding (2, 14). Regurgitation secondary to hiatal hernia and megaoesophagus has also been rarely described in canine tetanus (28–30). Vomiting and regurgitation are often confused one with the other by owners (even with targeted history taking) and thus it remains unclear, which of the two complaints was present in this cohort. In this study, vomiting/regurgitation was associated with non-survival. Four of 10 non-survivors (and none of the survivors) suffered from aspiration pneumonia and 3 of those 4 dogs had vomiting/regurgitation, implying the role of vomiting/regurgitation in the pathogenesis of this respiratory complication, which has clearly been related to death in this and previous research (12). Vomiting/regurgitation could have occurred as a consequence of gastric stasis, indicating disturbed autonomous function. Indeed, all non-survivors with the history of vomiting/regurgitation received atropine, suggesting the presence of autonomic dysfunction in these dogs. Gastric stasis has been shown to increase the risk of aspiration in critically ill dogs (31), and in tetanus, concurrently raised intra-abdominal pressure due to the spasm of abdominal muscles might further aggravate this risk (32). Interestingly, all 3 non-survivors with aspiration pneumonia and vomiting/regurgitation also had trismus. Inability to expel vomit from the mouth might have contributed to aspiration in those cases, the risk of aspiration being potentially further exacerbated by gastric tube feeding, which all of them received. Aspiration pneumonia might be the underlying mechanism for the association between trismus and non-survival in the present cohort of dogs. Furthermore, trismus might also be a sign of more advanced disease. Although it is a Class I sign (facial sign compatible with tetanus), it was in fact present in 8/29 of Class I and II dogs but in 9/12 Class III and IV dogs. Generally, trismus is likely to have a more serious impact on the patient (e.g., ability of food prehension) than other Class I signs such as wrinkled forehead or erect ears, which more or less represent an aesthetic problem rather than impacting any essential physiological processes.

In low-income countries, where access to high intensity care may be a challenge, prognosis of people with generalized tetanus is worse if clinical signs progress rapidly (4, 8, 9). In one study of people with tetanus, time from onset of specific signs to the first spasms of less than 3 days was associated higher mortality (9). This is largely consistent with the findings in our cohort, in which 7/10 non-survivors as compared to 11/32 survivors were ill for < 3 days prior to admission. However, there are reports of cases of canine tetanus complicated by autonomic dysfunction and/or need for mechanical ventilation, who deteriorated within 3–5 days after the onset of the first signs of tetanus but could be successfully managed and survived (14, 27).

This study also reviewed treatment modalities including surgical wound revision. Although a large meta-analysis including over 3,000 people with tetanus found shorter incubation periods (time from injury to the appearance of symptoms) in patients with wounds located at the head, and this in turn was associated with increased mortality (4), neither wound location nor surgical wound debridement were associated with survival in dogs in this or a previous report (2). It is, however, possible that published studies on canine tetanus did not reach statistical power to identify such association. Similar to other canine studies (2, 13), distal extremities were the most common sites or presumptive sources of infection. This is plausible, as these parts of the body have most contact to spores containing soil. In only a few dogs, the wound was located at the head (oral cavity in 3 and mandibular lymph node in 1 dog), and the site of infection was not identified in about one fourth of the cases, which, again, is similar to other reports (2, 3, 11). Based on their age, primary teeth eruption might have served as infection entry in at least 3 dogs where no wound was identified. In another dog, trivial wounds induced by fleas could have allowed entry of spores. Tick infestation with no evidence of other trauma has been reported in two dogs with tetanus (2, 33). Lack of association between surgical wound debridement and survival could implicate, that antibiotics are sufficient as a means to remove the source of infection. However, wounds positive for Clostridium tetani despite prolonged penicillin treatment (16 days) have been reported in humans (34), arguing in favor of surgery. Nonetheless, Sun et al. (10), discuss the possible negative impact of debridement (exacerbation of the symptoms of tetanus due to the intense stimulation caused by treatment of the wound itself), especially in cases of moderate to severe disease. They suggested, that wounds should only be debrided after the condition has stabilized.

Some controversy exists regarding the antibiotic of choice in patients with tetanus. Back in 1985, Ahmadsyah and Salim (35) demonstrated a survival benefit in moderately diseased patients treated with metronidazole as compared to procaine penicillin. However, in a recent prospective trial, outcome did not differ between patients treated with benzyl penicillin, benzathine penicillin or metronidazole, irrespective of disease severity (36). Likewise, multiple human and one canine study failed to find an association between antibiotic choice (mainly metronidazole and penicillin) and survival in tetanus (2, 6, 8, 10, 37), nor could such an association be identified in the study reported here. However, it is of note, that 78% (25/32) of survivors compared to only 30% (3/10) of non-survivors received antibiotics prior to referral, suggesting that early antibiotic treatment might be associated with favorable outcome. This is similar to cases of human and canine sepsis, where increased mortality has been reported in association with a delay in the administration of appropriate antibiotics (38, 39). Yet, our findings are in contrast with Burkitt et al.'s study, where no effect of timing of antimicrobial treatment on outcome could be identified, which the authors attributed to the small sample size. Another explanation for the association between timely usage of antibiotics and survival in this study might be very rapid progression of disease in non-survivors and therefore inadequate time to initiate antibiotic treatment. Forty-two percent of the dogs in the present study received two or more antibiotics during hospitalization. However, due to the retrospective nature of the study the indication for the use of multiple antibiotics could not be determined with certainty in all of these dogs. Seven dogs received a second or third antibiotic because of perioperative ampicillin usage (6 dogs) or evidence of sepsis (1 dog). In the remaining dogs the reason was unknown. In other case series proportion of those receiving two antibiotics was at least 30% (2, 3, 11) and prescription of more than two antibiotics is reported for aspiration pneumonia, urinary tract infections and the management of wounds on the basis of the results of culture and sensitivity tests (3, 14). Generally, in view of current antimicrobial stewardship guidelines, the use of multiple antibiotics needs to be considered carefully.

Although sedatives and muscle relaxants are an essential part of tetanus treatment, their effectiveness in treating canine tetanus or impact on survival have not yet been evaluated by objective methods, and the available information relies merely on anecdotal reports of individual cases. Acepromazine is commonly used in dogs with tetanus and some case reports or small case series suggested it might be more effective than benzodiazepines or methocarbamol to control spasms (11, 14, 40). In the present study, the use of this drug was associated with non-survival. The most likely explanation for this finding is that acepromazine was used to meet the demands of more severe disease. It is common practice at our institution to use diazepam as first line sedative in patients with tetanus. Only if this drug is not sufficient in controlling clinical signs, acepromazine and barbiturates might be added. This order of drug administration was described by other authors too (14), while some administered these sedatives the other way around (12). It should also be noted based on experimental data, that the ability of acepromazine to control tetanic spams diminishes with its repeated use and that tetanic activity can be exacerbated with increasing dosages of acepromazine (41). Whether such cumulative effect exists in clinical cases of canine tetanus is difficult to assess. If there is a cumulative effect on tetanic activity with acepromazine, one would expect the anticholinergic effect of this drug to cumulate as well. This, together with the vagolytic effect of atropine, the use of which also was more frequent in non-survivors, might have contributed to the pathogenesis of aspiration pneumonia, one of the major complications of tetanus, associated with increased mortality in this and a previous study (12). Atropine was shown to induce swallowing disorders and inhibit cough reflex even in low dosages (42). However, again, the use of atropine was likely indicated by the severity of the disease.

The effectiveness of methocarbamol as a muscle relaxant for controlling rigidity in dogs with tetanus has been questioned (2, 11). Our results are not in favor of its use either, but its effectiveness cannot be evaluated from a retrospective study. In human medicine methocarbamol has been replaced by benzodiazepines in the treatment of tetanus many years ago. Some authors suggest that lack of efficacy of methocarbamol might be due to its inability to control spasticity that originates from upper motor neuron injury (43). Clinical trials or experimental studies to support this opinion are lacking.

An interesting ancillary treatment for dogs with tetanus was presented in two recent case reports using magnesium constant rate infusion in supraphysiological dosages (27, 33). The authors expressed concerns about the depressive effect of high levels of sedation on the respiratory center, and claim that the use of magnesium allowed reduction in sedative medication (27). Indeed, in a large meta-analysis of human tetanus, higher sedative usage was associated with higher case-fatality (4). In the present study, the number of sedatives used per patient was not significantly associated with survival. However, the impact of oversedation cannot be assessed from retrospective data. Although oversedation could lead to increased risk of aspiration and cardiovascular and respiratory depression, undersedation, on the other hand can lead to uncontrollable tetany, pain, distress, hyperthermia (with its consequences such as coagulopathy or multiple organ dysfunction) and upper airway obstruction.

Many studies of canine tetanus directly or indirectly call into question the efficacy of tetanus immunoglobulin in dogs (2, 11, 13). The present study did not identify an association between antitoxin and survival either. One possible explanation for this could be, that in dogs unbound tetanospasmin, which is the substrate for antitoxin (1), plays a negligible role in the progression and outcome of the disease. Indeed, it is the amount of tetanus toxin that is irreversibly bound to CNS presynaptic sites, rather than the amount of circulating tetanospasmin, that determines how severe the course of the disease will be. The concept of efficacy of antiserum as a function of susceptibility to tetanospasmin is supported by the positive effects, that antitoxin exerts in more susceptible species, namely humans (4) and horses (23).

Tube feeding was not associated with survival and this matches directly (13) or indirectly (2, 12, 44) the results of former studies. By contrast, high caloric nutrition is reported to reduce the odds of death in people with tetanus (10). The lack of benefit of assisted feeding in canine tetanus might be due to the heterogenicity of disease severity in dogs included in the above-mentioned studies. Similarly, in a large retrospective analysis including 467 dogs suffering from various disease, energy supply indeed was positively associated with hospital discharge, but disease severity was the main negative predictor of outcome (45). Another explanation for the lack of effect of tube feeding on survival might be its relation to aspiration pneumonia, which might negate any potential effects of calorie supplementation. In dogs with tetanus, aspiration can occur antegrade due to accumulation of saliva in the pharynx, which tetanus patients are predisposed to due to dysphagia and hypersalivation. However, aspiration might also be caused by aspiration of gastric contents that reflux from the stomach into the pharynx. Nasogastric tubes have been shown to increase the risk of aspiration *via* loss of anatomical integrity of the upper and lower esophageal sphincters, increase in the frequency of transient lower esophageal sphincter relaxations, and desensitization of the pharyngoglottal adduction reflex (46). It has also been suggested that gastric bacteria could migrate upward along the tube and colonize the pharynx (46). One study in animal models (47) and another study in children (48) showed that gastrostomy tube placement might reduce lower esophageal sphincter (LES) pressure and increase the risk of gastro-esophageal reflux, with a change in the gastro-esophageal angle as the suspected mechanism. Sedatives also aggravate LES relaxation resulting in regurgitation of gastric contents (31). Sedatives are commonly used in dogs with tetanus and the use of some preparations (acepromazine, barbiturates) has been associated with non-survival in the present study, possibly by exacerbating risk of aspiration as discussed above. Given this possible association of assisted feeding with increased risk of aspiration but the previous positive effect of high-calorie nutrition in humans with tetanus, it could be of interest to evaluate the effect of total parenteral nutrition in future studies of canine tetanus. None of the dogs in the present study received parenteral nutrition. To minimize risk of aspiration, when feeding tubes are used in dogs with tetanus, gastric motility should be monitored closely and the use of prokinetic drugs should not be delayed when gastric stasis is detected. Also, the effect of sparing sedatives by use of magnesium to prevent gastroparesis should be investigated in future studies.

Complications are an important outcome measure in critical illness. Classification of complications is valuable to guide decision-making in clinical settings, aid client communication and allow for comparisons between studies. This study proposed classification of complications based on organ systems. Previous studies in dogs with tetanus did not classify complications based on organ systems and therefore direct comparison with our study is not possible. A recent canine tetanus study focussed on respiratory complications which occurred at a rate of 26% (14/53) (12). Lower prevalence of respiratory complications in the present report might be due to the high proportion of dogs with mild to moderate disease, as respiratory complications occurred in dogs with mean Burkitt et al.'s severity score of 3.2 (± 0.6) in the previous study (12) (this study defined Burkitt et al.'s score as continuous variable, which was normally distributed and therefore was reported as mean ± standard deviation). That study reported survival rate of approximately 14% (2/14) in dogs suffering from respiratory problems (12), which is comparable to our study, with only 1/5 dogs (20%) suffering from respiratory complications surviving. This dog was intubated to overcome laryngospasm and received short term ventilation. It was the only dog who received ventilatory support in our cohort and it could be speculated that ventilation might have improved outcome in other patients of respiratory failure. However, in dogs with pneumonia the use of ventilatory support has been associated with poor outcome (12, 49), and the remaining 4 dogs with respiratory complications in our study suffered from pneumonia. Gastrointestinal complications such as obstipation, gastric stasis and functional ileus were also frequent in the present study and were reported in both dogs (1, 2) and humans (32) previously. Some dogs suffered from several complications and fulfilled sepsis criteria (22). As we have classified complications based on affected organs systems, we have not specifically evaluated sepsis as complication. This condition is also difficult to diagnose from retrospective data. It is of note, however, that of dogs fulfilling sepsis criteria (*n* = 3), none survived. Generally, mortality from sepsis is reported to range from 31 to 100% depending on the number of dysfunctional organ systems (50).

In human medicine, in patients with access to ventilator, the presence of autonomic dysfunction is the main predictor of poor outcome (5, 7). According to Burkitt et al.'s classification scheme (2), dogs with autonomic derangements are automatically assigned to severity Class IV, which in Burkitt et al.'s as well as this study was associated with increased risk of death. Autonomic signs listed by Burkitt et al. include heart rate and blood pressure abnormalities but not body temperature derangements. In human tetanus, raised body temperature is considered a direct result of sympathetic overactivity (51). Therefore, hyperthermia, a clinical sign associated with non-survival in the present study, might in fact represent an autonomic system disturbance, raising the question if hyperthermia should be added onto the list of Class IV autonomic signs in Burkitt et al.'s scheme. On the other hand, the increased temperature might also be the result of tetanic spasms and the inability to loss heat through panting, which is one of the main mechanisms of heat dissipation in dogs. Therefore, hyperthermia might also have resulted from poor control of tetany and insufficient sedation. Irrespective of its cause, hyperthermia was more frequent in non-survivors in this study and in other reports of dogs with tetanus acute hyperthermia was documented to precede death (3, 12, 52).

Regarding autonomic signs, there was a predominance of sympathetic overactivity in the present study (tachycardia), similar to reports of experimental tetanus (53, 54) and some reports of naturally occurring disease (11). However, other studies predominantly found bradyarrhythmias, and episodes of enhanced sympathetic activity only occurred during tetanic spasms (2, 40). The fact that non-survivors in the present study received atropine more often than survivors indicates that bradycardia was present but was less pronounced than required to fulfill Burkitt et al.'s scheme criterion of ≤ 60 beats/min (2). The effect of vagolytic compound atropine might have masked the progression to bradycardic Class IV state in some cases. Taken together, it seems that the presence of autonomic disturbances, irrespective of its direction, indicates poor outcome.

This study has several limitations. The primary limitation is its retrospective design, with missing data and non-standardized treatment strategies and monitoring measures, which were left to the discretion of the attending clinician. In particular, continuous electrocardiogram and blood pressure monitoring were unavailable in many cases, therefore, the incidence of autonomic derangements might have been underestimated. Furthermore, occurrence of bradycardia might have been masked by frequent prophylactic use of atropine. It also needs to be taken into consideration that the present study included dogs referred to a university teaching hospital, which might potentially have introduced a selection bias toward more severe cases. Moreover, this was a single-center study. As treatment decisions and protocols likely differ between different institutions or veterinary practices, the findings might not be transferable to different cohorts of dogs with tetanus, treated at other facilities. However, at least the severity classification scheme of Burkitt et al. (2) seems to be widely applicable. Another important feature of veterinary studies in general is that euthanasia for non-medical reasons might negatively affect outcome, which has also been discussed in previous research of canine tetanus (12, 14). In the study reported here, euthanasia was the cause of death in 6/10 cases, with financial considerations playing a role in 2/6. Furthermore, it is possible, that the small sample size failed to identify a significant difference that actually existed between survival categories. On the other hand, as with all observational studies, it is possible, that associations with a certain treatment measure might have resulted simply from indication for its usage in more severe cases. All conclusions therefore must be interpreted on the grounds of this confounding factor. Another limitation is that only univariable analyses were performed for associations with survival; as such, it is possible that some of the factors found to be associated with survival are not independent survival predictors. However, our data can provide the basis for future prospective, adequately powered studies. These should aim to elucidate the prognostic significance of supraphysiologic magnesium treatment for its sedative sparing effects by using it from severity class II on (generalized tetanus) and of sub-staging Burkitt et al.'s scheme according to presence of hyperthermia, respiratory complications and whether or not dogs are fully grown (< vs. > 2 years of age). Given the low prevalence of tetanus in dogs, this research needs to be set up in a multi-center fashion.

In conclusion, the results of our study suggest, that young dogs, those with a rapid course of disease, those with respiratory complications and those with evidence of autonomic dysfunction are more likely to die of tetanus. Thereby Burkitt et al.'s classification scheme (2) is appropriate for prognostic assessment in its current version. However, modifications (e.g., integration of core temperature and presence of respiratory complications) might further improve its validity.

## Data availability statement

The raw data supporting the conclusions of this article will be made available by the authors, without undue reservation.

## Ethics statement

Ethical review and approval was not required for the animal study because it was a retrospective analysis. Written informed consent for participation was not obtained from the owners because it was a retrospective analysis.

## Author contributions

JZ collected and analyzed data and wrote the manuscript. JD contributed to data collection. JZ, AG, HL, and KH conceived the study. HL and KH edited the manuscript and both share senior authorship. All authors read and approved the final manuscript.

## References

[1] Greene CE. Tetanus. In:Sykes JE Greene C, editors. Infectious Diseases of the Dog and Cat. St. Louis, Missouri: Elsevier (2012). p. 423–31.

[2] Burkitt JM Sturges BK Jandrey KE Kass PH. Risk factors associated with outcome in dogs with tetanus: 38 cases (1987–2005). J Am Vet Med Assoc. (2007) 230:76–83. 10.2460/javma.230.1.76 17199496

[3] Adamantos S Boag A. Thirteen cases of tetanus in dogs. Vet Rec. (2007) 161:298–303. 10.1136/vr.161.9.298 17766808

[4] Woldeamanuel YW Andemeskel AT Kyei K Woldeamanuel MW Woldeamanuel W. Case fatality of adult tetanus in Africa: systematic review and meta-analysis. J Neurol Sci. (2016) 368:292–9. 10.1016/j.jns.2016.07.025 27538652

[5] Wasay M Khealani BA Talati N Shamsi R Syed NA Salahuddin N. Autonomic nervous system dysfunction predicts poor prognosis in patients with mild to moderate tetanus. BMC Neurol. (2005) 5:1–4. 10.1186/1471-2377-5-2 15679900 PMC548694

[6] Mahieu R Reydel T Maamar A Tadié JM Jamet A Thille AW. Admission of tetanus patients to the ICU: a retrospective multicentre study. Ann Intensive Care. (2017) 7:112. 10.1186/s13613-017-0333-y 29116572 PMC5676569

[7] Trujillo MH Castillo A Espana J Manzo A Zerpa R. Impact of intensive care management on the prognosis of tetanus. Analysis of 641 cases. Chest. (1987) 92:63–5. 10.1378/chest.92.1.63 3595250

[8] Saltoglu N Tasova Y Midikli D Burgut R Dündar IH. Prognostic factors affecting deaths from adult tetanus. Clin Microbiol Infect. (2004) 10:229–33. 10.1111/j.1198-743X.2004.00767.x 15008944

[9] Kosam DA. Clinical profile and prognostic indicators of tetanus in children. Int J Med Res Rev. (2015) 3:601–7. 10.17511/ijmrr.2015.i6.11724284024

[10] Sun C Zhao H Lu Y Wang Z Xue W Lu S. Prognostic factors for generalized tetanus in adults: a retrospective study in a Chinese hospital. Am J Emerg Med. (2019) 37:254–9. 10.1016/j.ajem.2018.05.039 29891121

[11] Bandt C Rozanski EA Steinberg T Shaw SP. Retrospective study of tetanus in 20 dogs: 1988-2004. J Am Anim Hosp Assoc. (2007) 43:143–8. 10.5326/0430143 17473020

[12] Guedra M Cortellini S Humm K. Respiratory complications in dogs with tetanus: a retrospective study of 53 cases. Can Vet J. (2021) 62:1202–6. 34728847 PMC8543695

[13] Shea A Hatch A De Risio L Beltran E. Association between clinically probable REM sleep behavior disorder and tetanus in dogs. J Vet Intern Med. (2018) 32:2029–36. 10.1111/jvim.15320 30315605 PMC6272037

[14] Low RM Lambert RJ Pesillo SA. Successful management of severe generalized tetanus in two dogs. J Vet Emerg Crit Care. (2006) 16:120–7. 10.1111/j.1476-4431.2005.00160.x

[15] Jackson CB Drobatz KJ. Iatrogenic magnesium overdose: 2 Case reports. J Vet Emerg Crit Care. (2004) 14:115–23. 10.1111/j.1534-6935.2004.00103.x

[16] Harvey ND. How old is my dog? identification of rational age groupings in pet dogs based upon normative age-linked processes. Front Vet Sci. (2021) 8:6–11. 10.3389/fvets.2021.643085 33987218 PMC8110720

[17] Bragg RF Bennett JS Cummings A Quimby JM. Evaluation of the effects of hospital visit stress on physiologic variables in dogs. J Am Vet Med Assoc. (2015) 246:212–5. 10.2460/javma.246.2.212 25554937

[18] Oncken AK Kirby R Rudloff E. Hypothermia in critically ill dogs and cats. Compend Contin Educ Pract Vet. (2001) 23:506–20. 10.1136/inpract.23.9.50610853283

[19] Penninck DG Nyland TG Fisher PE Kerr LY. Ultrasonography of the normal canine gastrointestinal tract. Vet Radiol. (1989) 30:272–6. 10.1111/j.1740-8261.1989.tb01799.x

[20] Foley P. Constipation, Tenesmus, Dyschezia, and Fecal Incontinence. In:Ettinger S Feldman E Cote E, editors. Textbook of Veterinary Internal Medicine. St. Louis, Missouri: Elsevier Saunders (2017). p. 633–8.

[21] Labato M. Pollakiuria, Stranguria, and Urinary Incontinence. In:Ettinger J Feldman E Cote E, editors. Textbook of Veterinary Internal Medicine. St. Louis, Missouri: Elsevier Saunders (2017). p. 666–72.

[22] Seymour CW Liu VX Iwashyna TJ Brunkhorst FM Rea TD Scherag A. AD. The third international consensus definitions for sepsis and septic shock (Sepsis-3). JAMA. (2016) 315:801–10. 10.1001/jama.2016.0288 26903338 PMC4968574

[23] van Galen G Rijckaert J Mair T Amory H Armengou L Bezdekova B. Retrospective evaluation of 155 adult equids and 21 foals with tetanus from Western, Northern, and Central Europe (2000–2014). Part 2: Prognostic assessment. J Vet Emerg Crit Care. (2017) 27:697–706. 10.1111/vec.12669 28960891

[24] Starybrat D Burkitt-Creedon JM Ellis J Humm K. Retrospective evaluation of the seasonality of canine tetanus in England (2006–2017): 49 dogs. J Vet Emerg Crit Care. (2021) 31:541–4. 10.1111/vec.13068 33960634

[25] Burkitt-Creedon JM Starybrat D Humm K. Lack of seasonality of canine tetanus in California. J Vet Emerg Crit Care. (2022) 32:840–1. 10.1111/vec.13240 36031746

[26] Matthews BR Forbes DC. Tetanus in a dog. Can Vet J. (1985) 26:159–61. 17422529 PMC1679988

[27] Simmonds EE Alwood AJ Costello MF. Magnesium sulfate as an adjunct therapy in the management of severe generalized tetanus in a dog. J Vet Emerg Crit Care (San Antonio). (2011) 21:542–6. 10.1111/j.1476-4431.2011.00674.x 22316201

[28] Acke E Jones BR Breathnach R McAllister H Mooney CT. Tetanus in the dog: review and a case-report of concurrent tetanus with hiatal hernia. Ir Vet J. (2004) 57:593–7. 10.1186/2046-0481-57-10-593 21851651 PMC3113811

[29] Van HL Van BH. Conservative treatment of tetanus associated hiatus hernia and gastrooesophageal reflux. J Small Anim Pract. (1992) 33:289–94. 10.1111/j.1748-5827.1992.tb01146.x

[30] Dieringer TM. Esophageal hiatal hernia and megaesophagus complicating tetanus in two dogs. J Am Vet Med Assoc. (1991) 1:87–9. 1885336

[31] Whitehead K Cortes Y Eirmann L. Gastrointestinal dysmotility disorders in critically ill dogs and cats. J Vet Emerg Crit Care. (2016) 26:234–53. 10.1111/vec.12449 26822390

[32] Cook TM Protheroe RT Handel JM. Tetanus: a review of the literature. Br J Anaesth. (2001) 87:477–87. 10.1093/bja/87.3.477 11517134

[33] Papageorgiou V Kazakos G Anagnostou T Polizopoulou Z. The role of magnesium in the management of acute and long-term symptoms caused by tetanus in two dogs. Top Companion Anim Med. (2021) 44:100535. 10.1016/j.tcam.2021.100535 33933700

[34] Campbell JI Lam TMY Huynh TL To SD Tran TTN Nguyen VMH. Microbiologic characterization and antimicrobial susceptibility of *Clostridium tetani* isolated from wounds of patients with clinically diagnosed tetanus. Am J Trop Med Hyg. (2009) 80:827–31. 10.4269/ajtmh.2009.80.827 19407132

[35] Ahmadsyah I Salim A. Treatment of tetanus: an open study to compare the efficacy of procaine penicillin and metronidazole. Br Med J. (1985) 291:648–50. 10.1136/bmj.291.6496.648 3928066 PMC1417474

[36] Ganesh Kumar AV Kothari VM Krishnan A Karnad DR. Benzathine penicillin, metronidazole and benzyl penicillin in the treatment of tetanus: a randomized, controlled trial. Ann Trop Med Parasitol. (2004) 98:59–63. 10.1179/000349804225003037 15000732

[37] Tosun S Batirel A Oluk AI Aksoy F Puca E Bénézit F. Tetanus in adults: results of the multicenter ID-IRI study. Eur J Clin Microbiol Infect Dis. (2017) 36:1455–62. 10.1007/s10096-017-2954-3 28353183

[38] Disselkamp M Coz Yataco AO Simpson SQ. POINT should broad-spectrum antibiotics be routinely administered to all patients with sepsis as soon as possible? Yes Chest. (2019) 156:645–7. 10.1016/j.chest.2019.05.030 31590706

[39] Summers AM Vezzi N Gravelyn T Culler C Guillaumin J. Clinical features and outcome of septic shock in dogs: 37 Cases (2008-2015). J Vet Emerg Crit Care. (2021) 31:360–70. 10.1111/vec.13038 33382202

[40] Panciera D Baldwin C Keene B. Electrocardiographic abnomalities associated with tetanus in two dogs. J Am Vet Med Assoc. (1988) 192:225–7. 3350751

[41] Laurence DR Webster RA. Tachyphylaxis to the antitetanus activity of some phenothiazine compounds. Br J Pharmacol Chemother. (1961) 16:296–308. 10.1111/j.1476-5381.1961.tb01088.x 13759492 PMC1482022

[42] Tsubouchi T Tsujimoto S Sugimoto S Katsura Y Mino T Seki T. Swallowing disorder and inhibition of cough reflex induced by atropine sulfate in conscious dogs. J Pharmacol Sci. (2008) 106:452–9. 10.1254/jphs.FP0071553 18344613

[43] Sibrack J Hammer R. Methocarbamol. (2022). Available online at: http://www.ncbi.nlm.nih.gov/pubmed/33351427 (accessed June 23, 2022).

[44] Löffler C. Tetanus beim Hund. Kleintier Konkret. (2015) 18:34–43. 10.1055/s-0035-1547399

[45] Brunetto MA Gomes MOS Andre MR Teshima E Gonçalves KNV Pereira GT. Effects of nutritional support on hospital outcome in dogs and cats. J Vet Emerg Crit Care. (2010) 20:224–31. 10.1111/j.1476-4431.2009.00507.x 20487250

[46] Gomes GF Pisani JC Macedo ED Campos AC. The nasogastric feeding tube as a risk factor for aspiration and aspiration pneumonia. Curr Opin Clin Nutr Metab Care. (2003) 6:327–33. 10.1097/01.mco.0000068970.34812.8b 12690267

[47] Canal DF Vane DW Goto S Gardner GP Grosfeld JL. Reduction of lower esophageal sphincter pressure with stamm gastrostomy. J Pediatr Surg. (1987) 22:54–7. 10.1016/S0022-3468(87)80015-5 3819994

[48] Grunow JE Al-Hafidh AS Tunell WP. Gastroesophageal reflux following percutaneous endoscopic gastrostomy in children. J Pediatr Surg. (1989) 24:42–5. 10.1016/S0022-3468(89)80298-2 2723992

[49] Lemieux E Rozanski E Buckley G Chalifoux N Kennedy C Lynch A. Indications and outcomes for puppies undergoing mechanical ventilation: 59 cases (2006 to 2020). Can Vet J. (2021) 62:839–42. 34341595 PMC8281947

[50] Kenney EM Rozanski EA Rush JE Delaforcade-Buress AM Berg JR Silverstein DC. Association between outcome and organ system dysfunction in dogs with sepsis: 114 cases (2003-2007). J Am Vet Med Assoc. (2010) 236:83–7. 10.2460/javma.236.1.83 20043806

[51] Hörtnagl H Brücke T Hackl JM. The involvement of the sympathetic nervous system in tetanus. Klin Wochenschr. (1979) 57:383–9. 10.1007/BF01480476 37368

[52] Rubin S Fauklner R Ward G. Tetanus following ovariohyterectomy in a dog: a case report. J Am Anim Hosp Assoc. (1983) 19:293–8.

[53] Sofola OA Odusote KA. The effects of induced hypoxia on the cardiovascular system in dogs poisoned with tetanus toxin. Naunyn Schmiedebergs Arch Pharmacol. (1982) 318:220–4. 10.1007/BF00500483 7063047

[54] Odusote KA Sofola OA. Haemodynamic changes during experimental tetanus toxicity in dogs. Naunyn Schmiedebergs Arch Pharmacol. (1976) 295:159–64. 10.1007/BF00499449 995212

