# Supplementary material for: Survival in canine tetanus – retrospective analysis of 42 cases (2006–2020)
Source: Front Vet Sci. 2022 Dec 15;9:1015569. doi: 10.3389/fvets.2022.1015569 (PMC9797805; doi:10.3389/fvets.2022.1015569)
Supplement: Supplementary file 1 [file Table_1.docx]

Suppl 1 Tetanus severity classification system for dogs adapted from Burkitt et al. (2007).

| **Class** | **Clinical signs*** |
| --- | --- |
| I | Any or all of the following: |
|  | - Miosis, enophthalmos, risus sardonicus, erect ears, or trismus - Hypersensitivity to noise, light, or touch - Ambulatory |
|  | Absence of any class II, III or IV signs |
| II | May include any or all class I signs |
|  | Any or all of the following: |
|  | - Dysphagia - Stiff gait, sawhorse stance, or erect tail - Ambulatory |
|  | Absence of any class III or IV signs |
| III | Must have class I or class II signs (requirement) |
|  | Any or all of the following: |
|  | - Recumbency - Muscle fasciculations or spasms - Seizures |
|  | Absence of any class IV signs |
| IV | Must have class I, II, or III signs (requirement) |
|  | Any or all of the following: |
|  | - Bradycardia (heart rate ≤ 60 beats/min) or bradyarrhythmia - Sinus tachycardia (heart rate ≥ 140 beats/min) or tachyarrhythmia - Labile hypertension (mean arterial blood pressure ≥ 130 mm Hg or systolic arterial blood pressure ≥ 150 mm Hg) - Labile hypotension (mean arterial blood pressure ≤ 60 mm Hg or systolic arterial blood pressure ≤ 80 mm Hg) - Periods of apnea or respiratory arrest |
| *Note that dogs graded as class II need not have class I signs, but dogs in class III must have class I or II signs. Dogs graded as class IV must have class I, II, or III signs. | |
